# Strong host phylogenetic and ecological effects on host competency for avian influenza in Australian wild birds

**DOI:** 10.1098/rspb.2022.2237

**Published:** 2023-01-25

**Authors:** Michelle Wille, Simeon Lisovski, David Roshier, Marta Ferenczi, Bethany J. Hoye, Trent Leen, Simone Warner, Ron A. M. Fouchier, Aeron C. Hurt, Edward C. Holmes, Marcel Klaassen

**Affiliations:** ^1^ Sydney Institute for Infectious Diseases, School of Life and Environmental Sciences and School of Medical Sciences, The University of Sydney, Sydney, NSW 2006, Australia; ^2^ WHO Collaborating Centre for Reference and Research on Influenza, at the Peter Doherty Institute for Infection and Immunity, Melbourne, VIC 3000, Australia; ^3^ Department of Microbiology and Immunology, University of Melbourne, at the Peter Doherty Institute for Infection and Immunity, Melbourne, VIC 3000, Australia; ^4^ Centre for Integrative Ecology, Deakin University, Geelong, VIC 3217, Australia; ^5^ Geelong Field and Game, Geelong, VIC 3340, Australia; ^6^ Wetlands Environmental Taskforce, Field and Game Australia, Seymour, VIC 3660, Australia; ^7^ Agriculture Victoria Research, AgriBio Centre for AgriBioscience, Bundoora, VIC 3083, Australia; ^8^ Department of Viroscience, Erasmus Medical Centre, Rotterdam 3015GE, The Netherlands; ^9^ Victorian Wader Study Group, Thornbury, Victoria 3071, Australia; ^10^ Australasian Wader Studies Group, Curtin, ACT 2605, Australia

**Keywords:** avian influenza, influenza A virus, host range, host susceptibility, host–pathogen dynamics, phylogenetic effects

## Abstract

Host susceptibility to parasites is mediated by intrinsic and external factors such as genetics, ecology, age and season. While waterfowl are considered central to the reservoir community for low pathogenic avian influenza A viruses (LPAIV), the role of host phylogeny has received limited formal attention. Herein, we analysed 12 339 oropharyngeal and cloacal swabs and 10 826 serum samples collected over 11 years from wild birds in Australia. As well as describing age and species-level differences in prevalence and seroprevalence, we reveal that host phylogeny is a key driver in host range. Seasonality effects appear less pronounced than in the Northern Hemisphere, while annual variations are potentially linked to El Niño–Southern Oscillation. Our study provides a uniquely detailed insight into the evolutionary ecology of LPAIV in its avian reservoir community, defining distinctive processes on the continent of Australia and expanding our understanding of LPAIV globally.

## 1. Introduction

Wild birds are believed to be the reservoir for most influenza A viruses and have been detected across more than 100 avian species [1]. Avian influenza viruses are predominately low pathogenic avian influenza A viruses (LPAIV) with limited signs of disease [2]. However, following spill-over into poultry, avian influenza virus may become highly pathogenic resulting in morbidity and mortality, thus causing substantial economic losses [3,4]. There is also continued concern about zoonotic transmission of avian influenza virus from poultry against the background of a continuously growing global poultry market [5,6]. A hallmark of this growing problem is spillback of highly pathogenic avian influenza virus into wild birds, which results in mass mortality events in wild birds and the global spread of these viruses [7].

Many pathogens such as LPAIV persist in multi-host systems, making the identification of infection reservoirs crucial for devising effective interventions. Yet, empirical characterization of host reservoir communities is a challenge [8]. Through intensive surveillance, members of the avian order Anseriformes, notably the family Anatidae (ducks, geese and swans), and to a lesser extent Charadriiformes (shorebirds and gulls) with emphasis on the family Scolopacidae (sandpipers), have been identified as key reservoirs of LPAIV [1]. Across sampled host species within these taxa, there appears to be significant heterogeneity in competency (here defined as the combined effect of exposure and susceptibility [9]), viral diversity and host response to AIV [1]. Indeed, ducks of the genus *Anas* have generally been reported to have high prevalence and diversity of AIV subtypes [1]. This has led to an overrepresentation of select host taxa, including *Anas* ducks, in research and surveillance systems.

In the light of this bias, it is important to recognize that our current understanding of LPAIV ecology is described from a duck-centric, particularly mallard-focussed (refer to electronic supplementary material, table S2 for scientific names), temperate and Northern Hemisphere perspective [10–13]. However, a continental-scale study across North America demonstrated that LPAIV infection dynamics vary due to differences in climate, seasonality and host ecology [14], with low-latitude environments having lower AIV prevalence with limited seasonal variation [14,15]. Data from Australia have shown low prevalence, no consistent seasonal pattern [16,17] and a profound inter-annual variation in the timing and quantity of rainfall strongly linked with El Niño–Southern Oscillation (ENSO) and Indian Ocean Dipole (IOD) [18–22]. Beyond Anseriformes, LPAIV dynamics and ecology in Scolopacidae is unclear, with low prevalence and haphazard sampling globally, with the exception of Delaware Bay, USA [23–26].

Taken together, we have a biased understanding of LPAIV ecology, with a strong focus on *Anas* ducks as reservoirs, and only a limited appreciation of geographical variations in these dynamics. Herein, we aim to address a number of key questions arising from this bias. First, to reveal the extent to which host species exhibit phylogenetically conserved patterns of LPAIV prevalence and seroprevalence, from which we can infer patterns of host competency [27,28]. Species-level differences in prevalence are often reported in AIV studies and are reflected by the various traits associated with both susceptibility (e.g. age, body condition and pre-existing immunity), and exposure (e.g. foraging behaviour, migration and reproduction) [9,29]. While factors such as age are well-established traits in susceptibility, the role of host phylogeny as a driver of these species differences has only rarely been considered [29] and has never been incorporated at either high (i.e. among avian orders) or low (i.e. within families) levels of classification. Second, while controlling for these potential host phylogenetic and phylogenetic-independent species effects, we revisit the effects of age, season and eco-region as key ecological factors known to play a role in LPAIV prevalence, particularly in a geographical and climatic region that has seen limited research into AIV ecology. We address these questions based on the analysis of more than 10 000 samples collected within a single study spanning 11 years and across 76 species and seven avian orders, allowing for both a broad and an in-depth phylogenetic comparison across a wide host landscape for this virus. Critically, we leverage both virological and serological data into our framework. While virological data are central to understanding active infection, it may be deficient when sampling sporadically or without prior information on timing, age or species to target. As such, the addition of serological data allows us to garner a more complete picture of LPAIV dynamics on this unique continent.

## 2. Methods

### (a) Sample collection and screening for low pathogenic avian influenza A viruses

Samples were collected between November 2010 and March 2021. Three main catching techniques were employed. Both oropharyngeal and cloacal samples were collected from each individual bird using a sterile tipped applicator and placed into virus transport media. Blood samples were collected from each bird, except for the hunted ducks. Up to 200 µl was collected, primarily from the brachial vein, using the Microvette capillary system for serum collection (Sarstedt). Samples were screened as previously described [30]. A number of PCR-positive samples generated in this study were subtyped and sequenced, and were incorporated into an Australia-wide multi-institution study [31]. Subtype information for the samples reported both in this study and in [31] are provided in the electronic supplementary material, table S1. More detail pertaining to capture, sampling handling, screening and subtyping methods is provided in the electronic supplementary material methods.

### (b) Data analysis

For oropharyngeal and cloacal swab samples collected separately, we considered an individual bird positive if either the oral or cloacal sample was positive and merged into a single entry.

We used phylogenetic generalized linear mixed effect models to investigate the simultaneous effects of species as a random variable and fixed-effect, explanatory variables age, eco-region, season and year on LPAIV prevalence and seroprevalence. For species, we evaluated both the phylogenetic species effect, which evaluates the contribution of shared evolutionary history among species (e.g. genetic factors; termed ‘phylogenetic effect’) as well as the species effect independent of the phylogenetic relationship between species (e.g. ecological factors; termed ‘species effect’). Bird age was presented in two categories: juvenile (i.e. hatch-year) or adult. Three eco-regions, i.e. temperate, arid and tropical, were used based on the 2012 Interim Biogeographic Regionalisation for Australia version 7 (https://www.environment.gov.au/land/nrs/science/ibra#ibra). Season was divided into summer (September–February) and winter (March–August). For migratory shorebirds, summer coincides with the arrival of birds from the breeding grounds followed by their primary moult. Winter includes the period of pre-migratory preparation prior to departure for Northern Hemisphere breeding grounds. This behaviour applies to birds in their second year and older; for most shorebird species birds in their first year remain in Australia for the southern hemisphere winter.

Species with fewer than 50 samples were excluded from the analyses. To evaluate phylogenetic and species effects across higher and lower levels of classification (i.e. comparing species across multiple orders versus a comparison of species within families), we ran analyses on three sets of taxa. First, a set containing all species sampled. Second, a subset of this first group with only species belonging to the family Anatidae and, third, only species belonging to the family Scolopacidae. For the latter two taxon sets, we removed year 2014, 2015 and 2016 and the tropical eco-region for Anatidae, and the arid eco-region for Scolopacidae, due to low sample sizes.

The analyses were conducted using Bayesian generalized (non-)linear multivariate multilevel models using the brm() function within R package brms (Bayesian Regression Models using ‘Stan’), using family ‘Bernoulli’ and default priors [32,33]. We ran a series of candidate models for each of the three taxon sets and for both LPAIV and seroprevalence, i.e. six model sets with 10 models each, following [27]: (i) a model containing only an intercept, (ii) a model containing an intercept plus the phylogenetic and species effects (iii) the full model containing all fixed-effect explanatory variables as well as the phylogenetic and the species random effects, (iv) the full model minus the phylogenetic effect, (v) the full model minus the species effect, (vi) the full model without phylogenetic and species effects, (vii) the reduced model, (viii) the reduced model without the phylogenetic effect, (ix) the reduced model without the species effect and (x) the reduced model without the species and phylogenetic effects. The reduced models included only the predictors found to be important, i.e. their 95% credible intervals (CIs) were non-overlapping with zero in the full models. Models were compared using their WAIC scores. Further data details pertaining to these models are provided in the electronic supplementary material, methods.

## 3. Results

### (a) Sampling regime

**Figure 1.**
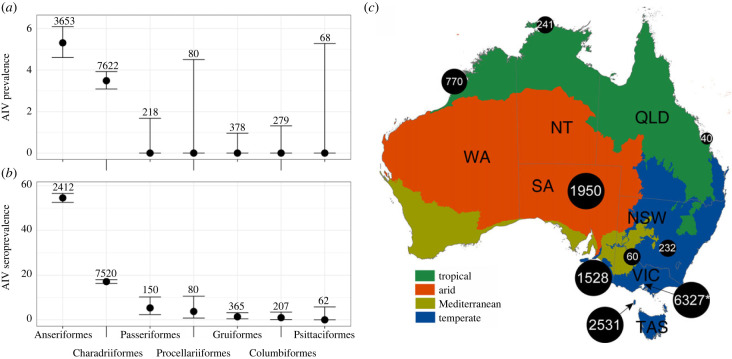
Sampling effort and virus prevalence across the study period. (*a*) Avian influenza viral prevalence based on qPCR of swab samples and (*b*) seroprevalence based upon a commercial anti-NP ELISA of serum samples. Points represent point estimates of percentage prevalence, and bars are the 95% confidence interval. Numbers above each estimate represent the number of samples included. For both (*a*) and (*b*), we excluded avian orders from which we had less than 10 samples collected throughout the entire study period. (*c*) Map illustrating geographical sampling effort across Australia. Map colours refer to eco-regions and was generated from https://www.environment.gov.au/land/nrs/science/ibra/australias-ecoregions, and distributed under a Creative Commons Attribution 3.0 Australia License. Herein ‘temperate’ includes both temperate grasslands and forests, ‘tropical’ includes tropical and subtropical forests and grasslands and ‘arid’ includes deserts and xeric shrublands. Australian state names are TAS—Tasmania, VIC—Victoria, SA—South Australia, NSW—New South Wales, WA—Western Australia, NT—Northern Territory and QLD—Queensland. Sampling location is indicated by a black circle, with size corresponding to the number of individuals sampled. Numbers within black circles refer to the number of individuals sampled; for some individuals, we may have both swab and serum samples and for others only swab or serum samples. Those samples collected from Victoria, but not in study sites in and around Melbourne have been added to the Victorian count, and this is indicated by an asterisk. A detailed breakdown of species composition is presented in the electronic supplementary material, table S2.

Between 2010 and 2021, 10 826 serum samples and 12 339 swab samples (combined oropharangeal and cloacal) were collected in Australia. The dataset comprises 11 orders, 25 families and 75 species of Australian birds, although the majority of the samples were collected from members of the family Anatidae within the Anseriformes (3657 swabs and 2412 serum samples) and family Scolopacidae within the Charadriiformes (7622 swabs and 7520 serum samples) (figure 1). Avian orders for which we had negligible sample numbers included the Galliformes (*n* = 4), Podicipediformes (*n* = 7) and Suliformes (*n* = 3) (electronic supplementary material, table S2).

Overall, we found evidence of LPAIV infection and anti-LPAIV antibodies in Anseriformes (5.4% virus prevalence and 53% seroprevalence) and Charadriiformes (3.5% virus prevalence and 17% seroprevalence), with 4% virus prevalence and 17% seroprevalence in the Scolopacidae. This is in accord with our expectation that members of these two orders (and families) of birds comprise the main LPAIV reservoirs. While we failed to find active LPAIV infection, we did detect low-level seropositivity in the Passeriformes (5.3%), Procellariiformes (3.8%), Gruiiformes (1.4%) and Columbiformes (0.97%). We found no evidence of anti-LPAIV antibodies in any of the 62 Psittaciformes tested (figure 1).

A total of 70 PCR-positive samples were subtyped through sequencing and include H1 (*n* = 2), H2 (*n* = 1), H3 (*n* = 11), H4 (*n* = 5), H6 (*n* = 9), H9 (=1), H10 (*n* = 17), H11 (*n* = 3), H12 (*n* = 18) and mixed (*n* = 3). The majority of subtyped/sequenced viruses are those collected from the Scolopacidae (*n* = 57), particularly ruddy turnstones sampled on King Island (*n* = 53), as compared to samples from the Anatidae (*n* = 13) (electronic supplementary material, table S1). Due to the extremely limited subtype data available, we have not integrated these data into further analysis in this study, but these data are integrated into an Australia-wide study of virus evolution [31].

To date, highly pathogenic avian influenza has never been detected in free-living wild birds in Australia [16,31].

### (b) Phylogenetic and non-phylogenetic species effects are key determinants of host competence

**Figure 2.**
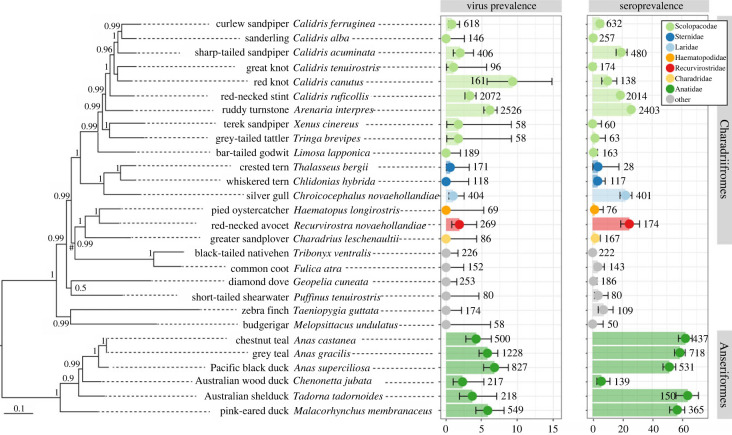
Prevalence and seroprevalence in Anseriformes and Charadriiformes. Host species are arranged taxonomically, according to maximum-likelihood phylogenies based on concatenated mitochondrial and one nuclear marker. Bayesian support values are presented at the node, and the scale bar indicates the number of substitutions per site. The hash symbol (#) refers to a node on the tree which does not conform to known phylogenetic relationships, and we were unable to resolve this discrepancy based on available host genetic data in GenBank. Species from which greater than 50 samples were collected are included, and percentage prevalence and 95% confidence intervals are plotted. Colours refer to host families in the order Anseriformes and Charadriiformes; host families from other orders are in grey. Sample size is plotted adjacent to the point estimate. Seroprevalence refers to the percentage prevalence of anti-NP antibodies in collected serum samples. Virus prevalence refers to the percentage prevalence of LPAIV by the use of qPCR. While we show seroprevalence for crested tern in this figure for clarity, it was not included in the seroprevalence all-species analysis due to low sample size (less than 50).

Six different species within the Anseriformes were included in our analysis. While this host order is considered central to the epidemiology of LPAIV and had an overall seroprevalence of 53% and virus prevalence of 5.4%*,* Australian wood duck was a clear exception with a substantially lower seroprevalence (2.8%) and viral prevalence (2.3%) compared to other duck species, suggesting that it is a less competent LPAIV host and as such plays a nominal role in AIV ecology (figure 2).

For the second most important host order for LPAIV, the Charadriiformes, we found marked heterogeneity in both seroprevalence and viral prevalence. For example, in the Scolopacidae family, we found higher viral prevalence and seroprevalence (greater than 10%) in ruddy turnstone, red nnot, sharp-tailed sandpiper and red-necked stint, with very low or no evidence of antibodies in bar-tailed godwit, great knot, curlew sandpiper and sanderling. The only other shorebird from which we detected LPAIV was red-necked avocet, family Recurvirostridae (figure 2). In addition to shorebirds, we also included three gulls and tern species. Viral prevalence was low (less than 1%) in all three species, although seroprevalence in silver gulls was 22.2% (figure 2), suggesting our sampling regime to detect AIV infection was possibly inadequate or NP-antibodies in this species are particularly long-lived.

Across all 10 candidate models tested for each of the three avian taxon sets, the models that considered phylogeny and species were the best fit for both virus prevalence and seroprevalence (i.e. had a ΔWAIC ≤ 2; table 1). Further, models that included all or a reduced set of explanatory variables, as compared to neither, greatly improved the performance of the candidate models in describing the three taxon sets across both virus prevalence and seroprevalence. As such, models including phylogenetic effects, species effects and other variables (such as age, eco-region, year and season) are required to adequately explain LPAIV variation.

**Table 1.** ΔWAIC values for all 10 candidate models, for both virus prevalence and seroprevalence in three different host taxon sets. Models that fit the data most satisfactorily (with a ΔWAIC ≤ 2) are italicized. Models are ranked based on overall performance, starting with models that tended to perform best in describing virus prevalence and seroprevalences across all three taxon sets. Generally, models that included both random effects (phylogeny and species), as well as a full or reduced set of fixed-effect, explanatory variables (age, eco-region, season and year) performed best in explaining the variation across all taxon sets, for both virus prevalence and seroprevalence.

model description					response ΔWAIC for prevalence in					
predictors			random effects		all species		Anatidae		Scolopacidae	
all	reduced	none	species	phylogeny	virus	serology	virus^c^	serology^c^	virus^a,b^	serology^b^
X			X	X	3.7	*0.6*	*1.3*	*0.0*	*0.2*	*1.7*
X				X	2.5	*0.7*	*0.4*	*0.3*	*1.2*	2.7
	X		X	X	*1.5*	*0.0*	3.3	6.9	*0.2*	*0.0*
	X			X	*0.0*	*1.1*	2.3	6.3	*1.1*	*1.4*
X			X		9.6	*0.9*	*0.7*	*0.2*	*0.1*	*1.4*
	X		X		7.2	*0.7*	3.4	6.8	*0.0*	*0.5*
		X	X	X	141.6	459.9	40.8	68.9	90.7	405.2
X					111.4	1529.3	*0.0*	135.2	32.0	396.3
	X				140.0	1529.1	2.1	149.6	30.1	445.3
		X			335.7	2367.3	44.0	242.4	169.8	830.2

^a^The models did not contain year as an explanatory variable, given poor model convergence when included.

^b^The models did not contain the arid eco-region due to low sample size.

^c^The models did not contain years 2014, 2015 and 2019, or the tropical eco-region due to low sample size.

**Figure 3.**
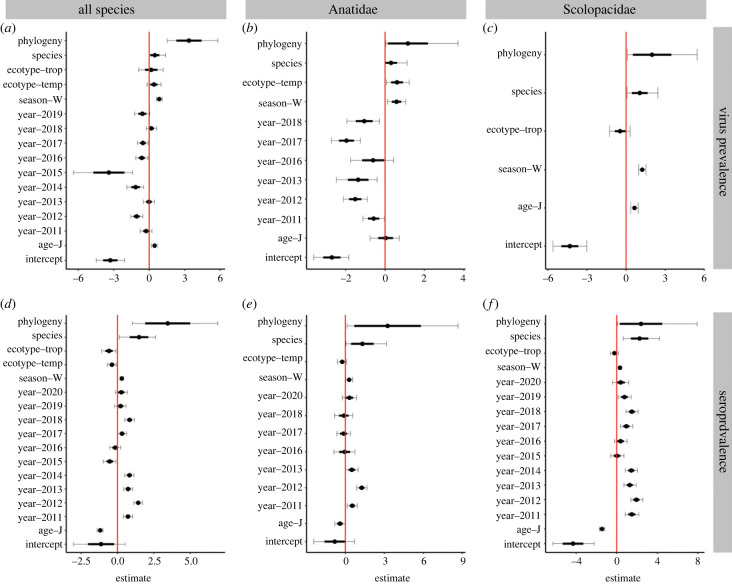
Posterior mean estimates with s.e. (thick bars) and 95% CIs (capped thin bars) of predictors and random effects on (*a–c*) LPAIV prevalence and (*d–f*) seroprevalence for (*a,d*) all species with greater than 50 samples, (*b,e*) Anatidae and (*c,f*) Scolopacidae for the full brms models. Parameters with intervals that do not overlap zero (indicated by a red line) are considered to have a significant influence on the response. Note that estimates are logits.

Considering all species, the phylogenetic signal, *λ*, which can vary between 0 (non-existent) to 1 (very strong) was generally strong in both viral prevalence (0.76) and seroprevalence (0.71; table 2 and figure 3). In addition to all species, we analysed the phylogenetic effect at two lower taxonomic levels (within the Anatidae and Scolopacidae). Within these key host families, the phylogenetic signals remained significant, varying between 0.27 and 0.60 (table 2 and figure 3). It is noteworthy that the phylogenetic effect at these lower (family) level comparisons was lower than when comparing species across the seven orders (table 2). However, it still showed that within these two major LPAIV host groups, considerable variation in competence levels exists between species. These species differences with a phylogenetic origin are further augmented by non-phylogenetic species differences, potentially related to differences in ecology, as evidenced by significant species signals among the top-ranking models in all taxon sets for both virus prevalence and seroprevalence (figure 3, table 1).

**Table 2.** Phylogenetic signal (*λ*) estimates with 95% CIs for full and reduced models with both phylogeny and species as random effects. Phylogenetic signals are only indicated if the model had a ΔWAIC ≤ 2.

	*λ* (95% CIs)	
	full model	reduced model
all species, virus prevalence		0.76 (0.47–0.91)
all species, seroprevalence	0.71 (0.24–0.94)	0.72 (0.25–0.93)
Anatidae, virus prevalence	0.27 (0.00–0.81)	
Anatidae, seroprevalence	0.60 (0.00–0.96)	
Scolopacidae, virus prevalence	0.45 (0.00–0.90)	0.42 (0.00–0.88)
Scolopacidae, seroprevalence	0.48 (0.00–0.95)	0.48 (0.00–0.95)

### (c) Seroprevalence and viral prevalence have inverse relationships with bird age

Across the four explanatory variables investigated, age was an important predictor of virus prevalence and seroprevalence in the models covering all species, with the Scolopacidae and the Anatidae showing a similar tendency (figure 3; electronic supplementary material, figure S1*a*). Across all species combined, juveniles had a 2.0% higher viral prevalence (95% CI 0.7–3.5%) and a 15.5% (−17.0 to −13.7%) lower seroprevalence as compared to adults (where percentages are calculated from the logit estimates depicted in figure 3). For the Scolopacidae only, these differences were 1.2% (0.5–2.0%) and −1.0% (−1.1 to −0.9%), for virus prevalence and seroprevalence, respectively. At a species level, significant differences in prevalence and seroprevalence between adults and juveniles were limited to species in which prevalence levels were also high for Scolopacidae (i.e. those species with a seroprevalence > 18%): red-necked stint, ruddy turnstone and sharp-tailed sandpiper (electronic supplementary material, figure S1*b*). Trending in a similar direction, in Anatidae, there was no significant age effect in virus prevalence (0.2%, 95% CI −3.2 to 5.8%) but there was in seroprevalence (–8.0%, −14.5 to 0.0%). At the species level within the Anatidae, there were no species where virus prevalence for juveniles was different from adults (electronic supplementary material, figure S1*c*). However, for both Pacific black duck and pink-eared ducks, the seroprevalence estimates for juveniles were lower as compared to adults (electronic supplementary material, figure S1*c*). Unfortunately, sample size for juvenile Anatidae was generally low (less than 50, with the exception of grey teal for which we had 66 samples), which may have played a role in only detecting a limited age-dependant effect on prevalence and seroprevalence for this avian family. As prevalence for avian species not in the Anseriformes or Charadriiformes was negligible (0% virus prevalence and 11% seroprevalence) no age-dependant patterns can be inferred for these orders.

### (d) Season and year modulate low pathogenic avian influenza A viruses prevalence and seroprevalence

Although less pronounced than northern hemisphere studies, season significantly affected prevalence levels in our data. Across all three species groups, winter viral prevalence was significantly higher compared to summer viral prevalence estimates (where again percentages are calculated from the logit estimates depicted in figure 3; all-species: 4.4%, 2.8–6.5; Anatidae: 4.4%, 0.8–9.8; Scolopacidae 3.2%, 2.1–4.6). Similarly, the same pattern was found in seroprevalence across all three species groups (all-species: 6.1%, 3.3–8.7; Anatidae: 6.5%, 1.1–12.7; Scolopacidae 0.5%, 0.2–0.8). In the case of Scolopacidae, summer includes the arrival of birds from their Arctic breeding grounds while winter includes birds sampled during the pre-migration phase.

In all three taxon groups, sampling year drove significant variation in virus prevalence, except for Scolopacidae, wherein the model including year did not converge. Given strong year effects for both the Anatidae (virus prevalence and seroprevalence) and Scolopacide (seroprevalence only), it is unsurprising that there was also a strong year effect in the all-species models. Based on the findings of [18], we compared annual rainfall across the Murray-Darling Basin (electronic supplementary material, figure S2), which covers most of southeast Australia, with the year effect estimates in virus prevalence in Anatidae (figure 3*b*), and found a significant correlation (*r* = 0.782, *N* = 7, *p* < 0.04). Within the Anatidae and all-species model, for which we can assess both virus prevalence and seroprevalence, we found no correlation between the pattern of virus prevalence (*r* = −0.228, *p* = 0.623) and seroprevalence (*r* = 0.430, *p* = 0.215) across years. That is, we did not observe high virus prevalence in years of low seroprevalence. We also found that the year effects in seroprevalence are different between Anatidae and Scolopacidae (*r* = 0.621, *p* = 0.100).

### (e) Role of eco-region in low pathogenic avian influenza A viruses prevalence

While the vast majority of our dataset comprises samples collected in temperate Australia, 1950 samples were collected in arid Australia (largely Anatidae) and 1062 samples were collected in tropical Australia (largely Scolopacidae). Interestingly, we only observed significant effects of eco-region on virus prevalence in Anatidae and on seroprevalence in the all-species taxon set. In the Anatidae*,* virus prevalence was higher in temperate regions as compared to arid regions (where percentages are calculated from the logit estimates depicted in figure 3; 11.0%, 6.7–18.5).

## 4. Discussion

Variation in host competence, through differences in exposure and susceptibility, among host species is a common feature of multi-host systems [27,28,34]. Our holistic study of LPAIV evolutionary ecology in wild birds is unique for its use of a paired virological and serological dataset, from which we inferred host competence and the role of host phylogeny in the ecology of LPAIV. A major advantage of the inclusion of serological data is that it expands the window of detectability of LPAIV. While LPAIV infections in individuals are only 7–11 days [10], anti-AIV antibodies may persist from months to years [35,36] and therefore population level seroprevalence is modulated by processes over much longer timescales like season, rainfall and migration [9]. The use of serology allowed us to identify bird taxa that, while they are unlikely to be important reservoirs, are occasionally infected by LPAIV (e.g. zebra finch). Adding serology thus also adds credibility that the results of viral prevalence data are not influenced by missing prevalence peaks and non-representative sample collection. This would manifest as low LPAIV prevalence but higher seroprevalence. In our dataset, silver gull might be an example of that, although the observation in this species could also be caused by exceptional long-lived anti-AIV antibodies. Conversely, where LPAIV prevalence matches seroprevalence levels, this may suggest unbiased sampling. For instance, both low viral prevalence and seroprevalence in sanderling and Australian wood duck as compared to other related taxa, suggests that those results are not likely to be biased by our sample collection regime and that these two species are probably true outliers within these two LPAIV-reservoir species groups. Across all species combined and within the more traditional hosts (Anseriformes and Charadriiformes), analysing both serological data and virology data strengthened the interpretation of the various random and fixed-effect explanatory variables, yielding largely overlapping and mutually supporting patterns.

Despite variations in prevalence reported across species in waterfowl or shorebird systems (e.g. [1]), the phylogenetic relationships among host species have never previously been integrated into statistical approaches to understand this variation in host prevalence, with the exception of [29]. The strong phylogenetic effect found in the all-species comparison is unsurprising given the identification of Anseriformes and to a lesser extent Charadriiformes as highly competent LPAIV hosts compared to other bird taxa decades ago [1,37]. However, the finding of a strong phylogenetic effect within both the Anatidae and Scolopacidae is striking. That phylogeny is such an important covariate strongly suggests that genetic relatedness, perhaps including shared aspects of the immune response and/or virus susceptibility, are at play. The strong phylogenetic effects identified may also be key elements of host–virus coevolution, and likely explain differences in host responses to infection, such as avoidance, resistance or tolerance. Indeed, it has long been argued that wild birds and LPAIV have undergone long-term coevolution, such that reservoir taxa may have adapted towards tolerance rather than resistance of LPAIV through mounting of a dampened immune response. In turn, (low pathogenic) LPAIV evolved low virulence in these hosts [2]. Indeed, Longdon *et al*. [28] and Barrow *et al.* [27], similarly infer that phylogenetic variation was driven by the generalized immune response, and that there has likely been long-term co-evolution between viruses/parasites and their hosts.

Beyond phylogenetic effects, species effects not driven by phylogeny appeared of importance. For instance, within the six *Calidris* species (i.e. curlew sandpiper to red-necked stint in figure 2), we found large variation in prevalence. These marked species differences across closely related species could be due to variations in habitat preference and degree to which they are associated with water. For example, sanderling is the most marine and beach-dwelling of all *Calidris* species. In addition, among the Anatidae*,* the most distantly related species (the *Anas* ducks versus the pink-eared duck) had similar prevalence values, whereas the intermediated related Australian wood duck had very low prevalence values. This is likely explained by foraging ecology, where Australian wood duck is an exclusive grazing duck in contrast with the other species that dabble or filter feed. Foraging ecology is suspected to play a key role in LPAIV ecology; the virus is transmitted by the faecal–oral route and avian taxa foraging in shallow water, such as members of Anseriformes and Charadriiformes play a central role in virus ecology. To date, there is only one study that has previously investigated the potential effect of specific species traits on AIV prevalence after correcting for phylogeny, which we have shown here to be crucial [29]. Having only a limited dataset at their disposal, they only found one weak effect across the eight ecological traits they investigated (migration distance). Obviously, the strong species effects found here warrant further investigation into which species traits, including foraging ecology, may explain differences in LPAIV prevalence between species.

This study uniquely describes a disease reservoir community and is the most comprehensive assessment of LPAIV ecology on the Australian continent. No previous studies have directly addressed host competency, age or eco-region, while only two studies addressed year and season effects [16–18,38,39]. First, in addition to species and phylogenetic effects, our statistical approach accounted for life history (age), seasonal, annual and environmental effects that are confirmed drivers of infection. As previously demonstrated, age is an important driver of LPAIV ecology. Higher LPAIV prevalence has been found in juvenile compared to adult ducks [10,11] and in mute swans the immune repertoire increases with age [36]. Second, as noted previously, seasonal cycle is central to prevalence: prevalence peaks are associated with autumn migration in the temperate north [10,11], with the arrival of European migrants in Africa [40,41] and with rainfall in Australia, although for the latter this is often on a multi-year rather than annual scale [18]. A determinative feature of the southern hemisphere climate, particularly Australia, is the ENSO and IOD linked irregularity in both timing and location of wet and dry periods [20]. As a result, breeding seasonality does not mirror that of northern hemisphere, rather some species may have elongated breeding times (5–7 months), or breeding may be competently opportunistic and take place at any time of the year with multiple broods in wet years [19,22]. Therefore, with increased rainfall, more juvenile birds are recruited into populations, driving an increase in the proportion of immunologically naïve birds in waterfowl populations, in turn modulating LPAIV prevalence [18]. Third, in addition to strong year effects associated with increased rainfall, we found that in the long-distance migratory Scolopacidae LPAIV prevalence was lowest just after their arrival from the breeding grounds and highest during the latter stages of the non-breeding season in Australia. Low population prevalence upon arrival may be due to parasites limiting migration [42] and thus new arrivals being preferentially free of pathogenic infections. Moreover, lower temperatures and lower UV levels during the latter stage of their Australian staging may be more conducive for virus survival [9]. Finally, despite the sampling across the three eco-regions arid, tropical and temperate being strongly biased towards the latter region, prevalence appeared lowest in arid environments, in line with the virus' susceptibility to desiccation [43].

Taken together, in addition to confirming the role of climate as well as (ENSO-linked) rainfall and age on LPAIV prevalence, we provide new insights into LPAIV evolutionary ecology that define the specific processes that occur on the continent of Australia and expand our understanding of the factors that modulate LPAIV ecology across wild birds globally. Notably the strong phylogenetic and non-phylogenetic species effects revealed here, highlight the importance of teasing apart these two overlooked factors in LPAIV ecology and evolution. Simultaneously considering the existence of strong phylogenetic and non-phylogenetic species effects, even within the two major LPAIV competent families, highlights how species-specific approaches are required in identifying reservoir communities, for understanding wildlife disease dynamics, and in evaluating spill-over risks from wildlife to livestock and humans.

## Data Availability

Underlying data are available in Dryad Digital Repository: https://dx.doi.org/10.5061/dryad.1zcrjdfv2 [44]. Data are provided in the electronic supplementary material [45].
